# An exploratory study on the placental efficiency, histomorphometric characteristics, hormonal shifts, and oxidative stress in dromedary camels with dystocia

**DOI:** 10.3389/fvets.2026.1750741

**Published:** 2026-03-02

**Authors:** Montaser Elsayed Ali, Ragab H. Mohamed, Fatma Ali, Amna H. M. Nour, Hassan A. Hussein, Mohamed Asran Elbehiry, Mohamed Abdelrahman, Fahad Alshanbari

**Affiliations:** 1 Department of Animal Productions, Faculty of Agriculture, Al-Azhar University, Assiut, Egypt; 2 Department of Theriogenology, Faculty of Veterinary Medicine, Aswan University, Aswan, Egypt; 3 Department of Physiology, Faculty of Veterinary Medicine, Aswan University, Aswan, Egypt; 4 Department of Zoology, Faculty of Science, Aswan University, Aswan, Egypt; 5 Department of Theriogenology, Faculty of Veterinary Medicine, Assiut University, Assiut, Egypt; 6 Department of Surgery, Obstetrics and Artificial Insemination, Faculty of Veterinary Medicine, Sphinx University, New Assiut, Egypt; 7 Department of Theriogenology, Faculty of Veterinary Medicine, Damanhour University, Damanhour, Egypt; 8 Department of Animal Production, Faculty of Agriculture, Assuit University, Assiut, Egypt; 9 Department of Medical Biosciences, College of Veterinary Medicine, Qassim University, Buraydah, Saudi Arabia

**Keywords:** delivery, dromedary camels, dystocia, eutocia, histomorphometry, placenta

## Abstract

The study investigated placental efficiency, histomorphometry, and hormonal concentrations (progesterone, estradiol 17β, and cortisol) alongside oxidative stress indicators (malondialdehyde (MDA) and total antioxidant capacity (TAC)) and performed correlation analyses among these parameters in eutocia, dystocia, and uterine inertia deliveries in dromedary camels. A total of 24 pregnant dromedary camels, aged 8–11 years, with an average body weight of 520 ± 75 kg, were categorized into three groups based on parturition outcome: the eutocia group (EG), *n* = 12; the dystocia group (DG), *n* = 5; and the uterine inertia group (UIG). *n* = 7. The results revealed significantly lower (*p* < 0.05) syncytiotrophoblast layer thickness, chorionic villi length and diameter, and syncytiotrophoblast layer integrity in dystocia deliveries. Additionally, the inflammatory cell infiltration was higher (*p* < 0.01) in DG than in UIG and EG. The placentas from EG had long, slender, and highly vascularized chorionic villi, while in DG placentas, the villi were shorter and atrophied. Progesterone concentrations were significantly higher (*p* < 0.01) in DG compared to UIG and EG. While DG had the lowest mean estradiol-17β concentration, EG had the highest. Moreover, MDA and TAC concentrations were lowest in the EG group, followed by the UIG group and then the DG group. Additionally, a positive correlation was found between chorionic villi diameter and placental efficiency, and between placental efficiency and vascular density. There were negative correlations among hormonal, antioxidant, placental efficiency, and histomorphometry parameters, as well as among these parameters and inflammatory cell infiltration. In conclusion, dystocia in camels was associated with hormonal dysregulation, presumably oxidative stress, and placental structural damage. However, for a definitive and powerful outline, further studies on large and more balanced populations are necessary in the future.

## 1 Introduction

The dromedary camel (*Camelus dromedarius*), also known as the Arabian camel, inhabits northern Africa, the Middle East, parts of Asia, and the Indian subcontinent (1). It is seasonally polyoestrous and an induced ovulation (2, 3), with a highly variable gestation period, ranging from 315 to 440 days (4). Although the incidence of dystocia in camels has been reported inconsistently in the literature, its reason remains unknown, with occurrence rates ranging from 2 to 9% (5, 6). Parturition disorders may be caused by changes in metabolic activity in the 3 weeks before and after parturition, primarily related to progesterone and estradiol 17β, or by an imbalance between the body’s antioxidant activity and increased lipid peroxide production and reactive oxygen species (7, 8). Indeed, in a variety of animal species, the fetal development is positively associated with the weight, size, and placenta quality as reflecting maternal nutrient supply and transporter abundance from the dam to the fetus, which is then reflected in birth weight (9–11).

The dromedary camel has a placenta that is classified into three structures: synepitheliochorial for the fetomaternal barrier, microcotyledonary for its villous arrangement, and zonary for its morphological shape (12). This specialized anatomy creates a broad, belt-like zone of intimate fetomaternal contact, thought to be an evolutionary adaptation for optimal nutrient and gas exchange in species facing metabolic challenges in arid environments (13). So, the placenta’s functional capacity is influenced by the microscopic architecture of microcotyledons, with key parameters such as surface area density, volumetric composition, and capillary vascularization being critical determinants of placental efficiency, defined as the mass of fetus produced per unit mass of placenta (14).

Furthermore, placental efficiency and nutrient transport are critical determinants of the fetus’s prenatal growth trajectory, affecting birth-related features such as birth weight, delivery mode, and litter size (15, 16). According to Noakes et al. (17), uterine inertia, also known as uterine atony, is the inability of the uterus to produce efficient contractions despite fetal positioning. The latter factors lead to prolonged or arrested labor, often causing obstetric intervention. Uterine inertia is rare in multiparous dromedary camels (18). Several bovine studies have explored hormonal and biochemical profiles, as well as placental structure and function (19, 20), but equivalent studies in dromedary camels are still to be documented.

Therefore, this study hypothesizes that hormonal imbalances, elevated oxidative stress, and placental structural abnormalities in dromedary camels contribute to dystocia, impairing fetal development and leading to difficult labor. So, this study analyzed histomorphometric changes, including syncytiotrophoblast thickness, chorionic villi structure, vascular density, and inflammation in dromedary placental tissues. Additionally, it looked to assess progesterone, estradiol 17β, and cortisol, as well as oxidative stress markers (MDA and TAC) under different delivery conditions, and investigate the relationships among hormone levels, oxidative stress, placental morphology, and delivery outcomes.

## 2 Materials and methods

### 2.1 Study location

The current study was conducted in several Egyptian villages in Aswan, located at 24° 05′20′′ N and 32° 53′59′′ E on the eastern bank of the Nile River. It is situated approximately 900 km south of Cairo, Egypt. All owners provided informed consent before the animals were included in the study.

### 2.2 Design and management

Twenty-four clinically healthy pregnant dromedary camels (*Camelus dromedarius*) with body condition scores of 3–3.5, an average body weight of 520 ± 75 kg (mean ± SD), and ages ranging from 8 to 11 years were enrolled in this study. The she-camels were pluriparous, exhibited typical clinical characteristics, and were naturally mated. Based on their parturition outcomes, the animals were classified into three groups: (i) eutocia group (EG, *n* = 12), She-camels that delivered calves spontaneously without human intervention, as defined by Hussein et al. (21). (ii) dystocia group (DG, *n* = 5), She-camels that experienced difficult deliveries requiring manual assistance (22, 23). (iii) uterine inertia group (UIG, *n* = 7) She-camels that delivered only after forced traction without manual corrections, according to (24). Furthermore, this data represents all cases observed and subsequently confirmed that met the inclusion criteria during the study period. Therefore, the imbalance reflects the observed birthing conditions during this period, not the result of a pre-designed distribution. The camels were maintained on a pasture-based diet, supplemented with alfalfa (*Medicago sativa*) when natural forage availability was limited. Fresh water was provided *ad libitum* throughout the study period. The first 10 days before the expected calving date, all signs of imminent parturition were closely monitored.

### 2.3 Placental efficiency

Placental efficiency was determined as the ratio of neonatal camel weight to placenta weight, expressed as a percentage, according to the following equation (25).


$$\text{Placental Efficiency}\;(\%)\;=\;\frac{\text{Neonatal camel weight}}{\text{Placenta Weight}}\;\times \;100$$

### 2.4 Histological study

Five full-thickness tissue blocks were obtained at random from the middle zone of each placenta’s belt, rinsed in sterile saline (0.9% NaCl), and immediately fixed in 10% neutral phosphate-buffered formalin (pH 7.0). Three non-overlapping, randomly selected microscopic fields (at 200x magnification) were examined from each block, for a total of 15 fields per animal. For histological processing, specimens underwent gradual dehydration through an ethanol series (50–99%), were cleared in methyl benzoate, and were embedded in paraffin wax at 58–62 °C. Serial sections (5 μm thickness) were cut using a rotary microtome and stained with Harris hematoxylin and eosin (H&E) for routine histological evaluation. Slides were examined under high-power light microscopy (Olympus BX43F, Tokyo, Japan). Digital image acquisition was performed using an Olympus DP74 camera (Tokyo, Japan). Histomorphological examination was performed by a specialist unfamiliar with the cohorts, and the fields were re-examined after 1 week to assess the accuracy of the measurements.

### 2.5 Biochemical assays

Blood samples were collected within 15 min. Postpartum, venous blood samples (*n* = 24) were collected from all animals via jugular venipuncture into plain serum tubes. Samples were centrifuged at 3000 × g for 20 min at room temperature, after which serum was aliquoted and stored at −20 °C until analysis. Serum concentration of progesterone and estradiol 17β was quantified using a chemiluminescent immunoassay (iFlash Immunoassay Analyzer, Shenzhen Yhlo Biotech, Co., Ltd., China). Cortisol concentrations were determined using a competitive ELISA (ABC Biotech kit, Berlin, Germany). Oxidative stress markers, total antioxidant capacity (TAC) and malondialdehyde (MDA) were determined using commercial colorimetric kits (Bio Diagnostic Egypt, CAT Nos. MD2529 and TA2513, respectively). Duplicate measurements were taken for all protocols, and each protocol was performed as per the manufacturer’s guidelines. In the method validation, the inter-assay coefficient of variation (%CV) was ≤10% for estradiol-17β, ≤8% for progesterone, ≤7% for cortisol, and ≤15% for some of the oxidative stress markers (MDA and TAC). Hormonal concentrations, particularly cortisol, are likely to be influenced by the acute stress associated with childbirth. Therefore, differences were compared between groups whose blood samples were collected simultaneously.

### 2.6 Statistical analysis

The statistical analyses were conducted using SPSS for Windows, Version 25 (IBM Corp., Chicago, IL, United States). The normality of the data distribution was assessed using the Kolmogorov–Smirnov test; data were considered normally distributed if *p* > 0.05. For comparisons among the eutocia, dystocia, and uterine inertia groups a one-way ANOVA was applied according to the general linear model:


$$Y_{\mathrm{ij}}=\mu +T_{i}+A_{j}+E_{\mathrm{ij}}$$

where Yij is the observed value, μ is the overall mean, Ti represents the treatment effect (delivery groups), and Eij is the random error. When ANOVA indicated significant differences, the Duncan *post-hoc* test was used for pairwise comparisons. Correlations between variables were evaluated using Spearman’s rank correlation coefficient (*ρ*), reported alongside 95% confidence intervals (CI) and corresponding *p*-values to convey both the strength and precision of the associations. A significance level of *p* < 0.05 was applied for all tests. R values are reported alongside their *p*-values to provide a direct measure of correlation strength.

## 3 Results

### 3.1 Neonatal calves’ weight, placental weight, and efficiency

The weight of neonatal calves and placentas, along with placental efficiency, was compared among camels experiencing eutocia (EG), dystocia (DG), and uterine inertia (UIG) in Table 1. Neonatal calves and placental weight (kg) were significantly higher (*p* < 0.01) in the DG group than in the EG and UIG groups. However, while placental efficiency did not differ significantly (*p* > 0.05) between the EG and UIG groups, it was significantly lower (*p* < 0.05) in the DG group than in either the EG or UIG groups.

**Table 1:** Neonatal camel weight, placenta weight and placenta efficiency in eutocia, dystocia and uterine inertia deliveries dromedary camels.

Item	EG	UIG	DG	*p*-value
Neonatal camel weight (Kg)				
Means	31.15^a^	30.44^a^	34.28^b^	0.01
SEM	1.09	0.47	0.22	
Placenta Weight (Kg)				
Means	3.78^a^	3.70^a^	4.34^b^	0.01
SEM	0.09	0.16	0.30	
Placenta Efficiency (%)				
Means	8.24^a^	8.22^a^	7.90^b^	0.05
SEM	0.24	0.21	0.17	

^a,b^Means with different superscripts in the same row are significantly different; EG, Eutocia group; UIG, Uterine Inertia group; DG, Dystocia group.

### 3.2 Histomorphometric analysis

Histomorphometric measurements of placental tissue in dromedary camels with eutocia (EG), dystocia (DG), and uterine inertia (UIG) are presented in Table 2. The syncytiotrophoblast layer thickness (μm) was significantly reduced (*p* < 0.05) in the DG group compared to both the EG and UIG groups. Additionally, chorionic villi length (μm), chorionic villi diameter (μm), and syncytiotrophoblast layer integrity were significantly lower (*p* < 0.01) in the DG group than in other deliveries. Furthermore, inflammatory cell infiltration (cells/field) was significantly higher (*p* < 0.01) in the DG group, while vascular density (vessels/10x field) was markedly reduced (*p* < 0.01) compared to the EG and UIG groups.

**Table 2:** Histomorphometric analysis of placental tissue measurements of eutocia, dystocia and uterine inertia deliveries dromedary camels.

Item	EG	UIG	DG	*p*-valu
Syncytiotrophoblast Layer Thickness (μm)				
Means	23.56^a^	22.37^a^	14.17^b^	0.05
SEM	3.35	4.20	3.81	
Chorionic Villi Length (μm)				
Means	186.82^a^	184.28^a^	126.37^b^	0.01
SEM	2.27	2.95	2.26	
Chorionic Villi Diameter (μm)				
Means	83.84^a^	81.37^a^	61.37^b^	0.01
SEM	9.29	10.95	9.16	
Syncytiotrophoblast Layer Integrity (%)				
Means	99.36^a^	98.92^a^	81.13^b^	0.01
SEM	3.39	2.48	6.15	
Vascular Density (vessels/10x field)				
Means	21.29^a^	19.74^b^	10.46^c^	0.01
SEM	1.92	2.17	2.83	
Inflammatory Cells (cells/field)				
Means	3.92^b^	3.34^b^	11.48^a^	0.01
SEM	1.01	0.92	2.48	

^a,b,c^Means with different superscripts in the same row are significantly different (*p* < 0.01).

### 3.3 Histological examination

**Figure 1:**
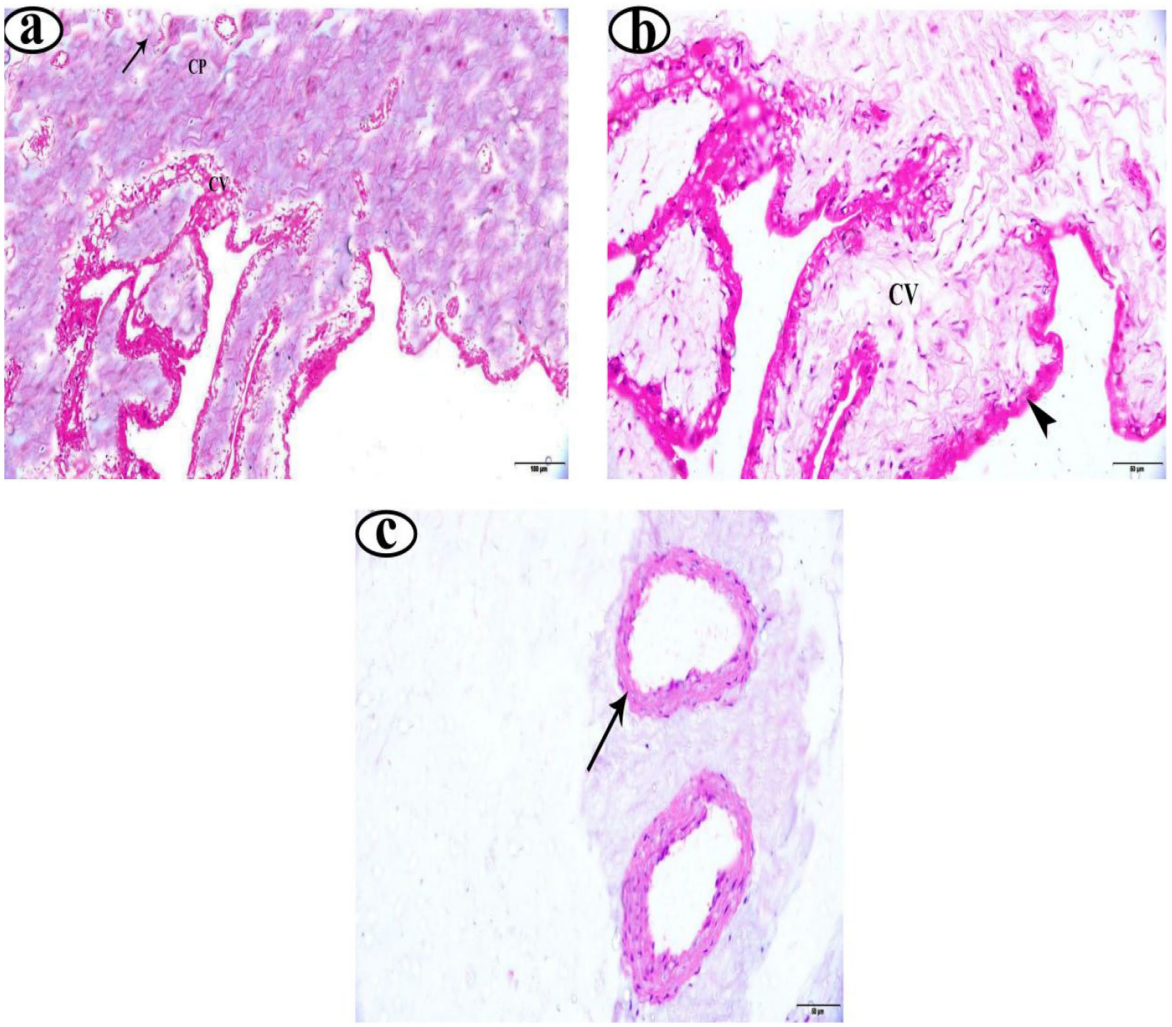
Photomicrographs of eutocia placenta stained with H&E **(a–c)**. Magnification 200X. Chorionic plate (CP), chorionic villi (CV), blood vessel (arrow), syncytiotrophoblast layer (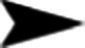).

**Figure 2:**
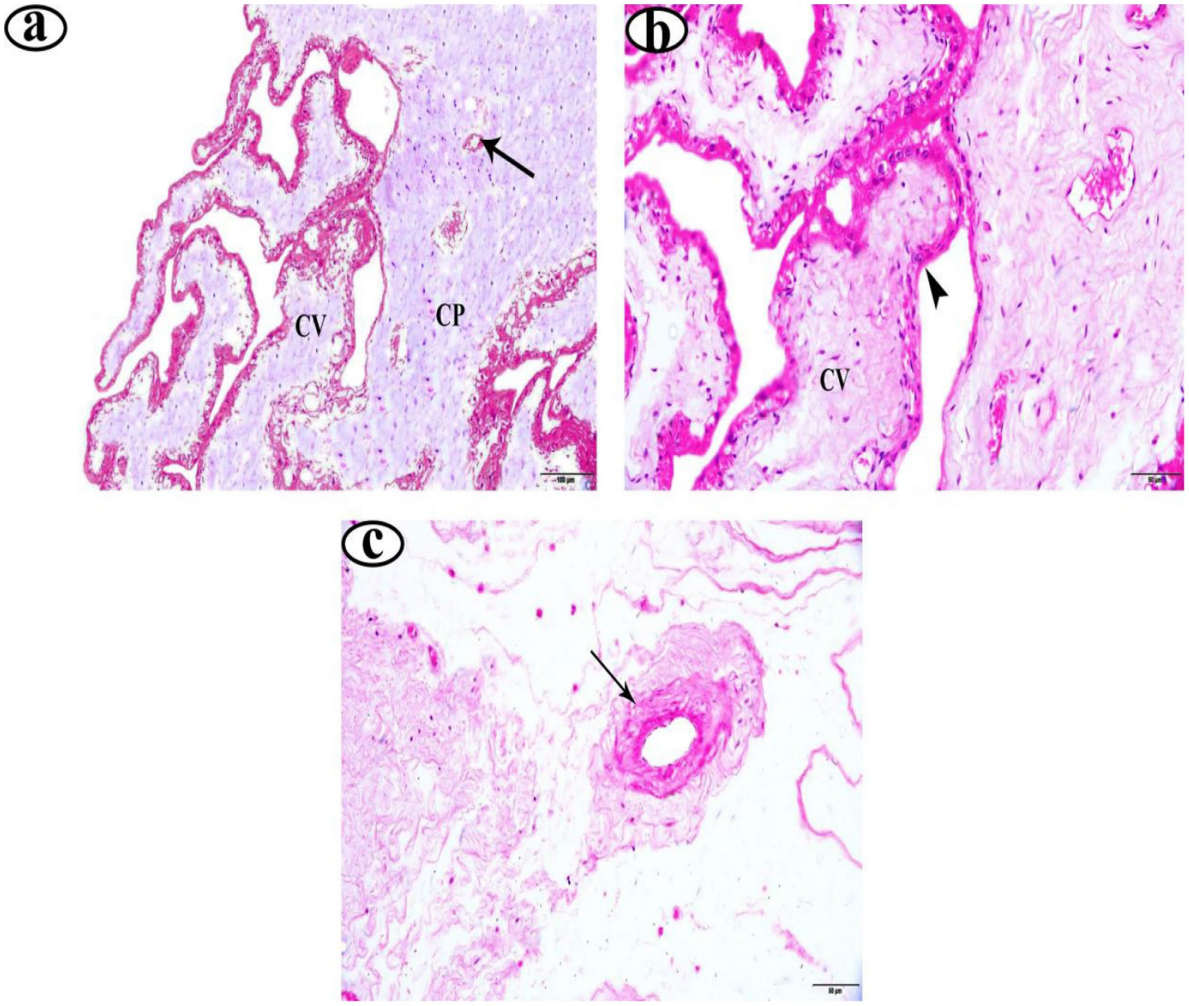
Photomicrographs of uterine inertia deliveries placenta stained with H&E **(a–c)**. Magnifications 200 X. Chorionic plate (CP), chorionic villi (CV), blood vessel (arrow), syncytiotrophoblast layer (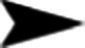).

**Figure 3:**
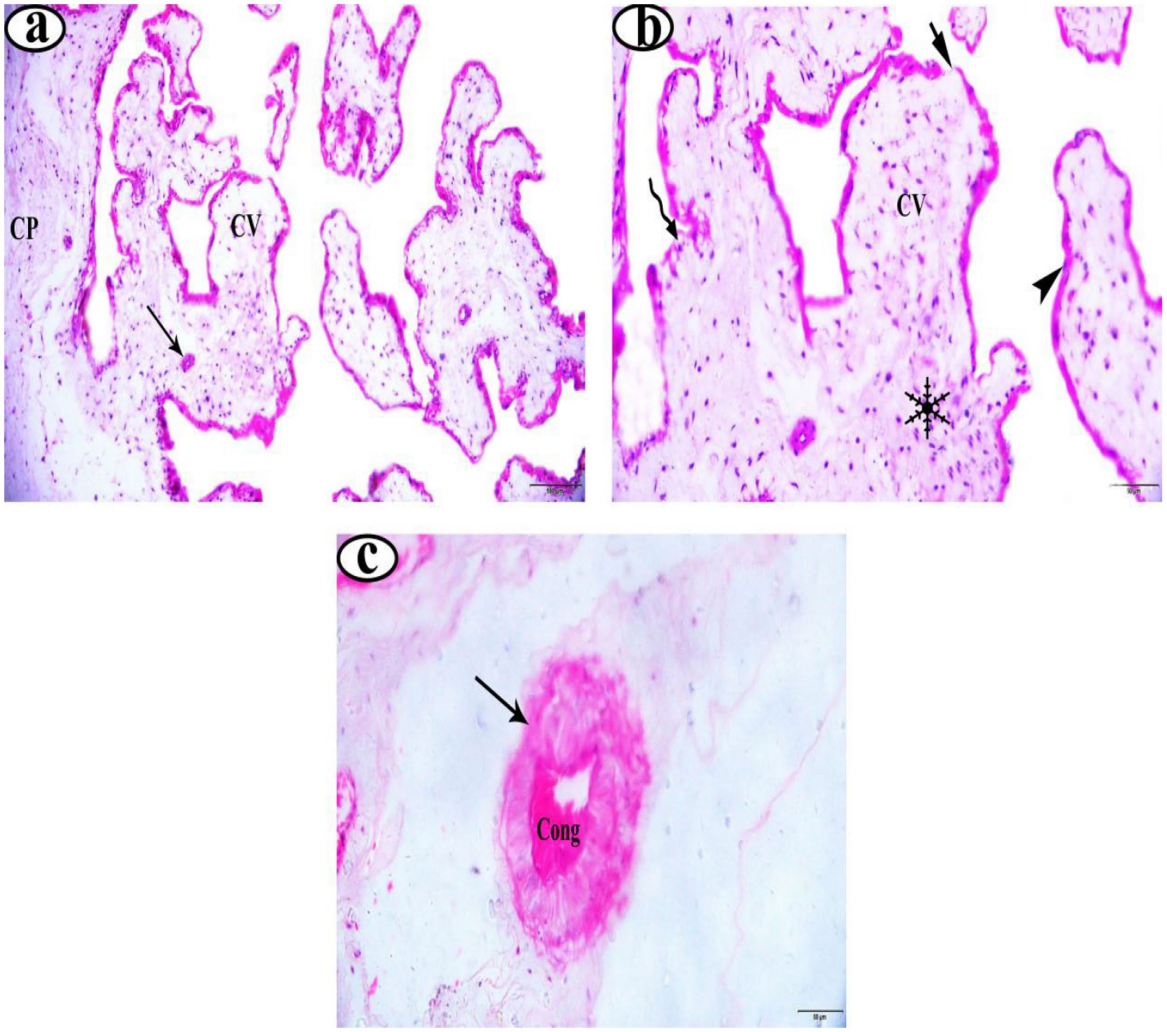
**(a–c)** Photomicrographs of in dystocia deliveries placenta stained with H&E. Magnifications 200X. Chorionic plate (CP), chorionic villi (CV), blood vessel (arrow), syncytiotrophoblast layer (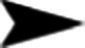), inflammatory cells (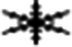), necrosis cells (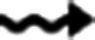), detachment areas (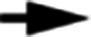), and blood vessel congestion (Cong).

In the EG group, placentas showed long, slender, and well-vascularized chorionic villi (Figure 1a). The syncytiotrophoblast layer appeared thick, intact, and well-organized (Figure 1b). Furthermore, the placentas from EG displayed higher vascular density, with many more well-formed blood vessels in the villous tissue (Figure 1c). Similarly, the UIG placentas group showed well-formed chorionic villi (Figure 2a) covered by an intact, uniformly structured syncytiotrophoblast layer (Figure 2b). The villous vasculature remained well-preserved, with no significant structural abnormalities (Figure 2c). In contrast, the DG placentas showed shortened, atrophic chorionic villi (Figure 3a) and a thin, irregular syncytiotrophoblast layer with focal necrosis, degenerative changes, and detachment. Additionally, the DG group tissues showed marked inflammatory infiltration (Figure 3b) and reduced vascular density, with visible vasoconstriction and partial vessel collapse (Figure 3c).

### 3.4 Correlation analysis of neonatal parameters and placental morphometric analysis

The data presented in Table 3 revealed that placenta efficiency showed no significant association with neonatal camel weight, but placental weight demonstrated a highly significant positive correlation with neonatal camel weight (*p* < 0.03; *r* = 0.579). Additionally, neonatal camel weight was significantly correlated with both placental weight (*p* < 0.03; *r* = 0.579) and syncytiotrophoblast layer thickness (*p* < 0.011; *r* = 0.509). Furthermore, placental efficiency exhibited a significant positive correlation with key histomorphometric parameters: syncytiotrophoblast layer thickness, chorionic villi diameter, vascular density, syncytiotrophoblast layer integrity, and vascular density. In contrast, inflammatory cell infiltration showed a highly negative correlation (*p* < 0.019) with placental efficiency (*r* = −0.475).

**Table 3:** Correlation analyses among new porn weight, placenta weight, placenta efficiency, and histomorphometric analysis in the eutocia, dystocia and uterine inertia deliveries dromedary camels.

Item	Neonatal camel weight (Kg)	Placenta weight (Kg)	Placenta efficiency (%)
Neonatal camel weight (Kg)			
*R*	--	0.579**	0.373
P	--	0.003	0.073
Placenta weight			
*R*	0.579**	--	0.536**
P	0.003	--	0.007
Placenta efficiency			
*R*	0.373	0.536**	--
P	0.073	0.007	--
Syncytiotrophoblast layer thickness (μm)			
*R*	0.509*	0.363	0.661**
P	0.011	0.081	0.001
Chorionic villi length (μm)			
*R*	0.307	0.198	0.590**
P	0.144	0.353	0.002
Chorionic villi diameter (μm)			
*R*	0.306	0.175	0.556**
P	0.147	0.412	0.005
Syncytiotrophoblast layer integrity (%)			
*R*	0.183	0.304	0.510*
P	0.393	0.148	0.011
Vascular density (vessels/10x field)			
*R*	0.354	0.448*	0.809**
P	0.089	0.028	0.001
Inflammatory cells (cells/field)			
*R*	−0.035	−0.225	−0.475*
P	0.872	0.289	0.019

*p, Correlation is significant at 0.05. **p, Correlation is significant at 0.01. r, Correlation value.

### 3.5 Progesterone, estradiol 17β, and cortisol concentrations

Progesterone, estradiol 17β, and cortisol serum concentrations in dromedary camels experiencing eutocia (EG), dystocia (DG), and uterine inertia (UIG) are shown in Table 4. Progesterone concentrations were significantly elevated (*p* < 0.01) in the DG group compared with both the EG and UIG groups. Additionally, the UIG group showed higher progesterone concentration (*p* < 0.01) than the EG group. Estradiol 17β concentrations were recorded at the lowest values in the DG group compared to other groups and at the highest values in the EG (*p* < 0.01). Cortisol concentrations differed significantly (*p* < 0.01) among groups.

**Table 4:** Progesterone, estradiol 17β, and cortisol concentrations (ng/ml) in the serum of eutocia, dystocia and uterine inertia deliveries dromedary camels.

Item	EG	UIG	DG	*p*-value
Progesterone concentrations				
Means	0.89^c^	1.46^b^	1.96^a^	0.01
SEM	0.11	0.09	0.02	
Estradiol 17β concentrations				
Means	749.63^a^	658.13^b^	546.88^c^	0.01
SEM	2.03	10.85	14.48	
Cortisol Concentrations				
Means	4.07^c^	4.79^b^	6.00^a^	0.01
SEM	0.12	0.15	0.06	

^*a,b,*c^Means with different superscripts in the same row are significantly different (*p* < 0.01).

### 3.6 Malondialdehyde and Total antioxidants

MDA and TAC concentrations in dromedary camels experiencing eutocia (EG), dystocia (DG), and uterine inertia (UIG) are shown in Table 5. The EG group showed the lowest (*p* < 0.01) MDA levels, with progressively higher levels in the DG and UIG groups. In contrast, the EG group showed the highest (*p* < 0.01) TAC levels, with the lowest in the DG group and intermediate in the IUG group.

**Table 5:** Malondialdehyde (MDA), and total antioxidants (TAC), in the serum of eutocia, dystocia and uterine inertia deliveries dromedary camels.

Item	EG	UIG	DG	*p*-value
Malondialdehyde (MDA), concentrations				
Means	22.70^c^	24.03^b^	25.14^a^	0.01
SEM	0.19	0.18	0.08	
Total antioxidants (TAC), concentrations				
Means	9.98^a^	9.01^b^	8.45^c^	0.01
SEM	0.08	0.05	0.14	

*^a,b,c^* Means with different superscripts in the same row are significantly different (*p* < 0.01).

### 3.7 Correlation analyses among hormonal, antioxidant, placental efficiency, and histomorphometric analysis

The data presented in Table 6 showed that the negative correlations (*p* < 0.01) among Progesterone concentrations and placental efficiency (*r* = 0.726), syncytiotrophoblast thickness (*r* = 0.879), chorionic villi length (*r* = 0.683), vascular density (*r* = 0.713), and syncytiotrophoblast integrity (*r* = 0.655), but there was a negative correlation (*p* < 0.01) with inflammatory cells. However, there was a significant positive correlation (*p* < 0.01) among estradiol 17β placental efficiency (*r* = 0.512), syncytiotrophoblast thickness (*r* = 0.807), chorionic villi length (*r* = 0.651), vascular density (*r* = 0.620), syncytiotrophoblast integrity (*r* = 0.631), and vascular density (*r* = 0.693). Placental efficiency, chorionic villi length, syncytiotrophoblast integrity, vascular density, and syncytiotrophoblast thickness all showed negative correlations with cortisol and MDA, contrary to TAC results.

**Table 6:** Correlation analyses among hormonal, antioxidant, placenta efficiency, and histomorphometric analysis in the eutocia, dystocia and uterine inertia deliveries dromedary camels.

Item	Progesterone (ng/ml)	Estradiol 17β (ng/ml)	Cortisol (ng/ml)	MDA	TAC
Placenta efficiency (%)					
*R*	−0.726**	0.512*	−0.728**	−0.604**	0.583**
P	0.001	0.011	0.001	0.002	0.003
Syncytiotrophoblast layer thickness (μm)					
*R*	−0.879**	0.807**	−0.817**	−0.820**	0.889**
P	0.001	0.001	0.001	0.001	0.001
Chorionic villi length (μm)					
*R*	−0.683**	0.651**	−0.736**	−0.687**	0.692**
P	0.001	0.001	0.001	0.001	0.001
Chorionic villi diameter (μm)					
*R*	−0.713**	0.620**	−0.678**	−0.599**	0.528**
P	0.001	0.001	0.001	0.002	0.008
Syncytiotrophoblast layer integrity (%)					
*R*	−0.655**	0.631**	−0.666**	−0.713**	0.579**
P	0.001	0.001	0.001	0.001	0.003
Vascular density (vessels/10x field)					
*R*	−0.795**	0.693**	−0.749**	−0.722**	0.686**
P	0.001	0.001	0.001	0.001	0.001
Inflammatory Cells (cells/field)					
*R*	0.627**	−0.593**	0.635**	0.615**	−0.518**
P	0.001	0.002	0.001	0.001	0.009

*p, Correlation is significant at 0.05. **p, Correlation is significant at 0.01. r, Correlation value.

## 4 Discussion

This study showed that dystocia in dromedary camels may induces significant histomorphometric alterations in placental tissues, disruption of steroid hormone and cortisol levels, and oxidative stress. These combined pathophysiological changes may negatively impact fetal growth and neonatal outcomes. The findings showed that perceived neonatal weight is significantly larger in both eutocia (EG) and uterine inertia (UIG) deliveries than in dystocia (DG) deliveries. These results support previous studies showing the association between dystocia and poor neonatal outcomes in ruminants (17). Additionally, placental weight was significantly higher in the DG and UIG groups than in the EG group, which could supply more nutrients and oxygen to the fetus prenatally (26). In the present study, DG and UIG showed shorter, narrower chorionic villi than the EG group. These findings were consistent with those of Voicu et al. (20), who documented that dystocia deliveries resulted in shorter, narrower chorionic villi, further supporting the notion that dystocia compromises placental development and function. The reduced size of the chorionic villi in dystocia may limit the efficiency of nutrient and oxygen exchange, potentially leading to fetal distress or breathing complications (27). This study found that the EG and UIG groups showed greater integrity of the syncytiotrophoblast layer than the DG group. In contrast, the DG group showed a significant reduction in syncytiotrophoblast layer integrity, which can compromise syncytiotrophoblast function (28). This loss of syncytiotrophoblast layer integrity could lead to suboptimal placental efficiency, contributing to poorer pregnancy outcomes, including compromised fetal wellbeing and potentially increased risk of stillbirth; this interpretation was in line with Wu et al. (29) and Fox et al. (30). The study reported that the vascular density in the EG and UIG groups was significantly higher than in the DG group. This increased vascular density may enhance nutrient and oxygen delivery to the fetus, thereby improving pregnancy outcomes (31). In this study, the placental efficiency measure supports species comparisons, but the Arabian camel’s unique placenta structure offers notable exploratory descriptive insights. The observed structural variations, particularly in the dystocia group, align with a poor efficiency index and indicate a histological trait associated with obstructed births in this species. The reduced vascular density observed in DG could be attributed to placental ischemia or inadequate remodeling of maternal blood vessels during complicated labor, hindering the efficient exchange of nutrients and oxygen between the mother and fetus (32). However, the number of inflammatory cells in the placental tissue was significantly higher in the DG group. This heightened inflammatory response can impair placental function and negatively change fetal health; these outcomes align with those of Kleiner et al. (25), who reported a high inflammatory response in dystocic animals.

There was an increase in progesterone and cortisol levels, and a decrease in 17β estradiol levels, in the DG and UIG groups compared to the EG group. This may contribute to labor disturbances, potentially leading to birth defects and reflecting either a cause or consequence of dystocia (33). Furthermore, the concurrent oxidative stress, as evidenced by higher MDA and lower TAC levels in the DG and UIG group compared to the EG deliveries, suggests significant free radical-mediated damage to placental tissues (34).

The study revealed negative correlations between progesterone concentrations and placental parameters. In this regard, a previous study (35) indicated that prolonged progesterone exposure may delay placental maturation and reduce nutrient transfer to the fetus. On the other hand, estradiol (17β) plays a crucial role in promoting trophoblast proliferation and angiogenesis, which is consistent with the positive correlations observed in our data between certain placental indices and this hormone (36). The results also showed a clear negative correlation between cortisol levels and placental morphological parameters, which is consistent with the literature describing the known negative impact of elevated stress hormones on placental structure (37).

Blood samples were obtained within 15 min postpartum, a period of rapid physiological variations, particularly in cortisol levels and other stress markers. As a result, judgments based on absolute differences in hormone levels in this study are purely descriptive, as the findings reveal intriguing association patterns that merit additional examination in future investigations.

### 4.1 Study limitations and comparative discussion

Although this study provides novel insights into placental biomarkers linked to dystocia in dromedary camels, it should be acknowledged that the small sample size and imbalance are inherent to the rarity and clinical nature of these cases. Despite these limitations, this work provides a crucial foundational description for future research. Dystocia is a significant medical and economic issue across various animal species. The Arabian camels range from 5 to 10%, compared to 15–20% of births requiring assistance in dairy cattle (38). Sheep have a higher incidence, especially in multiple pregnancies, where the rate reaches 80%, contrasting with just 5% for singleton births (39). In mare, dystocia is a critical emergency, with maternal mortality rates of up to 10% if not addressed promptly (40). Current physiological insights point to a shared pathological mechanism characterized by disruptions in maternal-fetal hormonal communication, which may lead to dystocia across different species (41).

## 5 Conclusion

This exploratory study concluded that there are presumbed associations between dystocia and uterine contractility in Arabian camels and indicators of placental insufficiency. These associations manifested as reduced placental efficiency, specific histological changes, and increased oxidative stress. To improve future outcomes, the study recommends enhanced prenatal care, including hormone monitoring, antioxidant supplementation, and early veterinary intervention. Due to the exploratory nature of the study and the small sample size resulting from the limited number of available dystocia cases, these results do not allow for causal inferences. Therefore, the study emphasizes the need for future studies with larger and more balanced populations to confirm these associations and explore the underlying causal mechanisms.

## Data Availability

The original contributions presented in the study are included in the article/supplementary material, further inquiries can be directed to the corresponding author.
