# Correction: An exploratory study on the placental efficiency, histomorphometric characteristics, hormonal shifts, and oxidative stress in dromedary camels with dystocia

**DOI:** 10.3389/fvets.2026.1835120

**Published:** 2026-04-07

**Authors:** Frontiers Production Office

**Affiliations:** Frontiers Media SA, Lausanne, Switzerland

**Keywords:** delivery, dromedary camels, dystocia, eutocia, histomorphometry, placenta

In the published article an incorrect version of affiliation 2 was included.

Affiliation 2. Department of Theriogenology, Faculty of Veterinary Medicine, Aswan University, Aswan was erroneously given as 2. Department of Theriogenology, Faculty of Veterinary Medicine, Assiut University, Assiut, Egypt.

Affiliation 2. Department of Theriogenology, Faculty of Veterinary Medicine, Aswan University, Aswan was omitted for author Ragab H. Mohamed. This affiliation has now been added for author Ragab H. Mohamed.

The authors list and the corrected affiliations list are appear below:

Montaser Elsayed Ali^1^, Ragab H. Mohamed^2^, Fatma Ali^3^, Amna H. M. Nour^4^, Hassan A. Hussein^5,6^, Mohamed Asran Elbehiry^7^, Mohamed Abdelrahman^8^ and Fahad Alshanbari^9*^

^1^ Department of Animal Productions, Faculty of Agriculture, Al-Azhar University, Assiut, Egypt

^2^ Department of Theriogenology, Faculty of Veterinary Medicine, Aswan University, Aswan, Egypt

^3^ Department of Physiology, Faculty of Veterinary Medicine, Aswan University, Aswan, Egypt

^4^ Department of Zoology, Faculty of Science, Aswan University, Aswan, Egypt

^5^ Department of Theriogenology, Faculty of Veterinary Medicine, Assiut University, Assiut, Egypt

^6^ Department of Surgery, Obstetrics and Artificial Insemination, Faculty of Veterinary Medicine, Sphinx University, New Assiut, Egypt

^7^ Department of Theriogenology, Faculty of Veterinary Medicine, Damanhour University, Damanhour, Egypt

^8^ Department of Animal Production, Faculty of Agriculture, Assuit University, Assiut, Egypt

^9^ Department of Medical Biosciences, College of Veterinary Medicine, Qassim University, Buraydah, Saudi Arabia

The original version of this article has been updated.

